# The importance of being second

**DOI:** 10.1371/journal.pbio.2005203

**Published:** 2018-01-29

**Authors:** The PLOS Biology Staff Editors

**Affiliations:** Public Library of Science, San Francisco, California, United States of America

Scientific research can be a cutthroat business, with undue pressure to publish quickly, first, and frequently. The resulting race to publish ahead of competitors is intense and to the detriment of the scientific endeavor. Just as summiting Everest second is still an incredible achievement, so too, we believe, is the scientific research resulting from a group who have (perhaps inadvertently) replicated the important findings of another group. To recognize this, we are formalizing a policy whereby manuscripts that confirm or extend a recently published study (“scooped” manuscripts, also referred to as complementary) are eligible for consideration at *PLOS Biology*.

Being scooped is loosely defined as when two independent groups studying the same system produce the same or similar results, and one group publishes their work first. Being scooped is often considered to devalue the second, complementary study; many journals will reject it citing lack of novelty. However, there is a self-evident benefit to publishing complementary research, and at *PLOS Biology*, we consider that two papers from two groups independently identifying the same phenomenon in parallel increase the confidence in the results of the work.

This new policy, acknowledging the value of complementary studies, therefore addresses the current concern regarding the reproducibility, or lack thereof, of scientific findings. Currently, the gold standard for demonstrating that an article is based on solid results is a replication study. These studies are generally conducted after publication and are considered critically important for supporting and advancing scientific theories. We argue that the “organic” replication of a complementary study is even better than a post-hoc and often costly replication study for supporting conclusions. There are other efforts underway to improve reproducibility and encourage replication, such as the Reproducibility Project: Cancer Biology (https://osf.io/e81xl/), as well as endeavors to implement high-quality reporting. With consideration of complementary research, *PLOS Biology* will support and promote scientific reproducibility and replication.

By formalizing this policy and providing a venue for complementary studies, *PLOS Biology* ensures high visibility for well-supported, significant research findings. We wish to recognize both the value of validating results and the researchers undertaking the work. Highlighting replication studies will ultimately prove positive for the public perception of science.

Although we are only now articulating our editorial policy regarding complementary research officially, we have implemented this policy on a case-by-case basis previously. Under our newly codified policy, authors of a complementary study have six months from the publication or posting (to a preprint server) of the first article to submit their manuscript to *PLOS Biology*. We hope that authors will use these six months to fully support and potentially extend the results of the first article. Complementary research submitted beyond the six-month period may still be considered, depending on individual circumstances. All submissions must still meet our editorial requirements for depth of study and potential impact.

By these means, we hope to promote replication and to provide a high-quality venue for these complementary studies. We welcome feedback from the community on this policy and our other efforts to strengthen the scientific literature. Please write to the editors at plosbiology@plos.org.

